# Examination of Cardiac Activity with ECG Monitoring Using Heart Rate Variability Methods

**DOI:** 10.3390/diagnostics14090926

**Published:** 2024-04-29

**Authors:** Galya Georgieva-Tsaneva, Evgeniya Gospodinova, Krasimir Cheshmedzhiev

**Affiliations:** Institute of Robotics, Bulgarian Academy of Science, 1113 Sofia, Bulgaria; jenigospodinova@abv.bg (E.G.); cheshmedzhiev@gmail.com (K.C.)

**Keywords:** cardiac monitoring, sensors, mobile device, ECG, HRV analysis, recurrence plot, Poincaré plot, 3D phase space attractor, statistical analysis, arrhythmia, syncope

## Abstract

The paper presents a system for analyzing cardiac activity with the possibility of continuous and remote monitoring. The created sensor mobile device monitors heart activity by means of the convenient and imperceptible registration of cardiac signals. At the same time, the behavior of the human body is also monitored through the accelerometer and gyroscope built into the device, thanks to which it is possible to signal in the event of loss of consciousness or fall (in patients with syncope). Conducting real-time cardio monitoring and the analysis of recordings using various mathematical methods (linear, non-linear, and graphical) enables the research, accurate diagnosis, timely assistance, and correct treatment of cardiovascular diseases. The paper examines the recordings of patients diagnosed with arrhythmia and syncope recorded by electrocardiography (ECG) sensors in real conditions. The obtained results are subjected to statistical analysis to determine the accuracy and significance of the obtained results. The studies show significant deviations in the patients with arrhythmia and syncope regarding the obtained values of the studied parameters of heart rate variability (HRV) from the accepted normal values (for example, the root mean square of successive differences between normal heartbeats (RMSSD) in healthy individuals is 24.02 ms, while, in patients with arrhythmia (6.09 ms) and syncope (5.21 ms), it is much lower). The obtained quantitative and graphic results identify some possible abnormalities and demonstrate disorders regarding the activity of the autonomic nervous system, which is directly related to the work of the heart.

## 1. Introduction

Patients diagnosed with cardiovascular diseases located in remote locations can be followed with remote monitoring, and all patients will have equal rights to health care along with the people living in large cities. Consultations with doctors from prestigious hospitals can also be conducted, which will lead to an increase in the effectiveness of the prescribed treatment and improvements regarding the health care and health status of the people.

The implementation of the remote monitoring of basic vital indicators of individuals enables the optimal, timely, and effective monitoring of patients with chronic risk diseases, as well as diseases that occur rarely but can be life-threatening.

People suffering from cardiovascular diseases are at high risk of contracting various types of viruses [1,2]. This is due to the weakened ability of the body (as a result of suffering from heart disease) to respond adequately to the disease.

Cardiac arrhythmias occur as a result of disturbances in the electrical impulses of the heart. Arrhythmias can be associated with various symptoms [3,4,5] and are expressed in a strong slowing of the heart rate (bradycardia), an excessive acceleration of the heart rate (tachycardia), or an irregular heart rate (fibrillation or flutter). Some types of arrhythmias can be harmless, but others can lead to serious complications in the activity of the heart. In some individuals, they can be completely asymptomatic, and the only way to recognize them is to examine the presence of disturbances in their heart rhythm. Scientific researchers have found that the heart rate and variability change [6] before the onset of arrhythmia, and, therefore, monitoring the variability in the real-world conditions in at-risk patients can serve to prevent some life-threatening situations.

One of the ways to reduce the risk of severe cardiovascular disease as a result of a virus is to strengthen the body’s defenses and regularly monitor the activity of the heart.

Syncope is a sudden and transient loss of consciousness that is associated with impaired cerebral blood supply, regarding which recovery may occur spontaneously. The seizures that occur during syncope can be dangerous and even life-threatening (for example, if the individual is driving at the time). For this reason, research into this specific disease can be useful in establishing its causes, treatment options, and prevention.

Cardiac or cardiovascular syncope is caused by various heart diseases such as bradycardia (sick sinus syndrome, conduction disorders, etc.), tachycardia (e.g., ventricular tachycardia), drug-induced arrhythmia, or certain types of hypotension.

Heart diseases with a violation of the structure of the heart are a serious cause of syncope and include valve diseases, heart attacks, heart tumors, etc. To correctly diagnose syncope and, accordingly, to carry out the correct treatment, it is necessary to conduct timely examinations, including ECG [7], echocardiography, computed tomography, coronagraphy, etc. This will lead to the administration of the appropriate treatment.

Heart rate variability is a time interval series that is derived from recorded ECG and PPG signals and is an established method for assessing the cardiac activity of the heart [8,9]. HRV is a non-invasive diagnostic method for assessing the internal dynamics in a heart rate interval series. The conducted research on heart rate variability shows that it changes under conditions of stress [10,11], professional sports activity [12], physical exercise [13], fatigue, anxiety [14], and depression [15]. The conducted studies [16,17] found reduced SDNN values and reduced parasympathetic nervous system activity after viral diseases.

In this study, the monitoring of vital signs is realized using a complex mobile sensor device for the simultaneous registration of ECG and photoplethysmographic (PPG) signals. Preprocessing takes place in the device itself, after which the processed data are analyzed using appropriate mathematical methods. In the event of the need for emergency medical intervention (in the case of a significant difference in the studied indicators, indicating a sharp change in the health status of the monitored individual), the device can send a signal to the monitoring doctor to provide emergency medical assistance.

### The Purpose of This Article

The main objectives of this article are as follows:

1. The study of the possibility of the quantitative assessment and visual display of cardiovascular activity when applying linear, non-linear, and graphic mathematical methods.
2. The evaluation of the effectiveness of the methods was conducted by applying statistical analysis in the study of three different cardiac conditions: healthy, arrhythmia, and syncope.

## 2. Materials and Methods

### 2.1. Device for Cardio Signals

**Figure 1:**
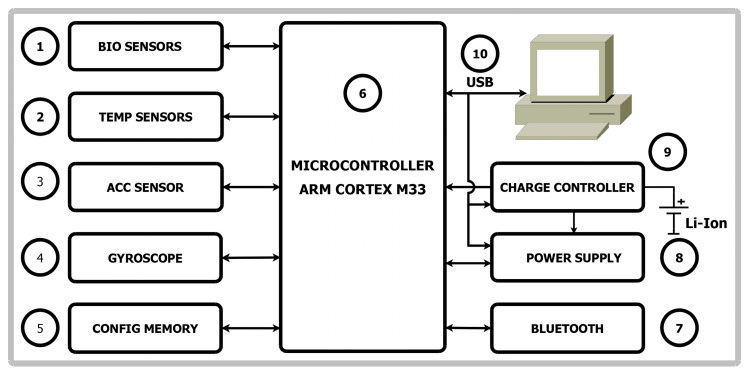
Block diagram of a device for cardio signal registration.

The created mobile sensor experimental device (Figure 1) enables the study of the body’s vital signs. EGG sensor, SPO2 reading sensor, and PPG sensor (operating on optical principle) can work simultaneously in continuous recording mode. Temperature sensors monitor the temperature of the environment and the body, as well as the body’s reaction to external temperature changes. The hardware solution includes an accelerometer and a gyroscope, enabling the device to detect a fall, and a prolonged state of rest–sleep, loss of consciousness, and movement. If a fall or loss of consciousness is detected (as in a syncope situation), a signal for help can be sent as soon as a continued lack of movement is detected. Using the thus-created device, ECG signals were recorded and analyzed in this paper.

The registered cardio signals undergo preprocessing directly in the mobile device itself using established procedures for reducing interference, removing artifacts, and correcting the drift of the baseline by sequentially applying an averaging filter, direct current (DC) component removing, and median- and low-pass filters.

The sensor system consists of the following elements:

1. Biosignal sensors including optical sensors for PPG and SPO2, single-lead ECG sensor.
2. Two temperature sensors—one for measuring body temperature and another for measuring ambient temperature.
3. Accelerometer—provides data on body movement.
4. Gyroscope—a sensor for determining the orientation of the body.
5. Configuration memory for settings, calibration constants, device operating modes, etc.
6. Microcontroller—a microcontroller with a Cortex M33 core (ST Microelectronics, Plan-les-Ouates, Switzerland) was selected with relatively large RAM (random-access memory)—2.5MiB—and nonvolatile memory (FLASH)—4MiB. This enables system expansion in the future.
7. For connection to phones, tablets, personal computers, etc., Bluetooth interface is used.
8. The power supply unit provides the necessary voltage and current for the operation of a system.
9. Charge controller for the built-in Li-ION/Li-POL battery.
10. A USB interface is used to connect to personal computers and charge the battery.

ECG registration through the created experimental device was performed through a single lead. A Microe ECG-6 Clik development board (ECG 6 Click (mikroe.com)-Microe, Beograd, Serbia) was used. Three electrodes are placed on the subject’s body and the heart’s activity is recorded, with the captured data saved in the device’s buffer memory.

For the preprocessing of the registered signals, C++ software (gcc 10.3.1) was created and implemented in the device. Preprocessing includes signal noise reduction (using linear filters to remove low-frequency and high-frequency noise components), detection of QRS complexes, creation of the RR interval time series, and creation of the normal RR interval (HRV) time series. For the detection of the QRS peaks, the frequently used Pan–Tompkins algorithm was applied, including the determination of the first derivative, squaring and integration (to reduce the probability of wrong recognition of the maximum amplitude deviations), thresholding, and decision rules.

Mathematical analyses are carried out on the HRV series created in this way using standardized linear methods and developing non-linear methods.

The created experimental device for recording cardio signals can be connected to a personal computer via a USB interface.

### 2.2. Analysis Methods Used

The linear methods used in the present study are widely used and standardized [8]. SDNN (mean of the standard deviations of all NN (normal_to_normal) intervals for all 5 min segments of the entire record), SDANN (standard deviation of 5 min average NN intervals), RMSSD (root mean square of successive differences between normal heartbeats), SDind (statistical) are defined. From the statistical geometric indices, HRV triangular index (HRVTi) and triangular interpolation of RR intervals (TINN) were studied. Frequency techniques are applied by fast Fourier transformation in three domains: Very Low Frequency (0.003–0.04 Hz, VLF), Low Frequency (0.04–0.15 Hz, LF), and High Frequency (0.15–0.4 Hz, HF). In this study, the absolute power spectrum components and the normalized units of measurement of the LF and HF components (normal units (n.u.)) are defined and compared. The LF/HF index was also calculated.

The following graphical methods were used in the study:

**Welch periodogram**

The Welch periodogram is a modification of the traditional periodogram and is among the most popular methods of spectral analysis. The studied time series is divided into M number of overlapping windows to reduce the high variance of the periodogram. Data located at the end of the time series receive a smaller weighting factor than data located in the center.

The modified periodogram is applied to all windows and the averaged spectral density is calculated [18]:

(1) $$Periodogram=\frac{1}{K}\sum_{i=0}^{K-1}P_{\mathrm{Modified},\;i}\left(f\right),$$

where:

K—number of blocks;
f—frequency;
i—index of the current window;
$P_{\mathrm{Modified},\;i}\left(f\right)$ is a modified periodogram for the i-th window of the time series [19]:

(2) $$Periodogram=\frac{1}{K.M.U}\sum_{i=0}^{K-1}{\left|\sum_{n=0}^{M-1}x_{i}\left(n\right).w\left(n\right)e^{-j2\pi \mathrm{fn}}\right|}^{2}$$

**Power spectral density**

The evaluation of the HRV in the frequency analysis [20,21] is carried out by determining the power spectral density (power spectral density—PSD) in the individual frequency areas. Cardiac recordings can be from 5 to 10 min, defined as short-term cardiac data recordings, and 24 h (or more) of cardiac data recordings, which are defined as long-term cardiac data recordings. There are some differences in how spectral analysis of short-term and long-term recordings of cardiac data is performed. In the present study, the investigations were conducted on a 40 min series of recorded data.

**Poincaré plot**

The Poincaré plot is a visual method that can be used for the analysis of heart rate variability in the field of cardiology, sports medicine, the assessment of various stress conditions on the cardiac health of people, as well as for scientific research. The main characteristics of the method are the following [22,23,24,25,26,27]:

- Heart rate visualization: The method provides a graphical visualization of heart rate variations. Each pair of RR intervals includes the current state RR(n) and the next state RR(n + 1) and is represented by a point in a rectangular coordinate system. Various states of cardiac activity can be identified by the shapes of the obtained graphic images. The chart in a healthy individual has the shape of a comet, while in an arrhythmia it is a fan, and in syncope a torpedo or consisting of several segments. Asymmetry of the image can provide information about non-uniformity in the heart rhythm and possible abnormalities.
- Assessment of the autonomic nervous system: The Poincaré plot can provide information on the balance between the sympathetic and parasympathetic nervous systems. This is important for understanding cardiac regulation and for assessing autonomic nervous system function. During physical exertion, mental stress, and cardiovascular diseases, the activity of the sympathetic nervous system increases, which is expressed by an increase in heart rate and a decrease in HRV.
- Quantitative assessment of heart rate variability [28,29,30]: The method offers quantitative parameters for HRV assessment, such as SD1 (Standard Deviation 1) and SD2 (Standard Deviation 2). These two parameters are a measure of short-term and long-term HRV and can be useful for assessing the risk of various diseases, monitoring the effectiveness of treatment, as well as for the balance between the sympathetic and parasympathetic nervous systems. Higher values of SD1 and SD2 are generally associated with greater cardiac variability and better health, while lower values may be indicative of abnormalities in cardiac or autonomic nervous system function. However, these parameters must be interpreted in the context of the individual patient and their accompanying factors and symptoms.
- Assessment of physiological responses [31,32,33]: People can be exposed to various physiological and mental stress situations, and Poincaré plot diagrams can show how the heart responds to them by reflecting changes in the time intervals between cardiac cycles.

**Recurrence plot**

A recurrence plot (RP) is a visual method for time series analysis and is used to detect repeating patterns or recurrent events in the studied data [34,35]. The basis of the method is the construction and analysis of a recurrence diagram (matrix), in which each point represents a comparison between two points in the time series. When constructing the chart, if a point (i, j) in the matrix is colored, it means that the states of the time series at times i and j are close or “repeating”. When points that are close in time form diagonal lines in RP, this indicates a recurrence between two points that appear in the time series with a certain interval or period. Some of the main characteristics of the recurrent method are the following [36,37,38,39]:

- Diagonal lines: Diagonal lines in the recurrence plot indicate the presence of periodic or repeating structures in the time series of the studied system. Longer diagonal structures indicate that there are more periodic or repeating segments in the system dynamics.
- Vertical and horizontal lines: These lines are indicators of various aspects of cardiac variability and can be useful in assessing heart health and autonomic nervous system function. The vertical lines in the recurrence plot indicate periods of stability in the heart rate. They are formed when the heart rhythm remains stable and is sustained over some time. A high frequency of vertical lines can be an indicator of good heart rate regulation and high heart variability. The horizontal lines in the image reflect the short-term changes in heart rate that can occur due to the presence of external factors such as stress, physical exertion, or emotional factors. These lines indicate that the heart rhythm is changing or adapting to external influences. A greater amount of horizontal lines can be an indicator of a greater sensitivity of the heart to external influences or disturbances in the regulation of the heart rhythm.
- Distribution of points: The distribution of points in a recurrence plot can provide information about the characteristics of the system’s dynamics, including equilibrium, chaos, or periodicity. A high concentration of points around the main diagonal of the RP indicates a repetition of certain events in the time series. This can be an indicator of stability or regularity in the time series. Scattered dots in RP may indicate randomness or chaos in the time series. If the points are evenly distributed throughout the matrix, this may indicate a lack of regularity in the time series. The presence of clusters or areas of a high density of points may indicate the presence of events or periods with similar characteristics or behavior in the time series.
- Shape and size: The shape and size of the recurrence graph can be analyzed to extract characteristics of the system, such as the degree of chaos, the degree of predictability, and others.

The numerical analysis of the recurrence plot includes the following parameters [40]:

- Recurrence rate (REC %)—This parameter provides quantitative information about the level of recurrence in the analyzed time series and can be useful for comparing different time series or for tracking changes in the structure of the time series over time. The parameter indicates the percentage of recurrent points in the chart, which is calculated by dividing the number of recurrent points that are on the main diagonal by the total number of points in the matrix. A higher REC value indicates greater density in the time series, which can be interpreted as a greater degree of structure or repeatability in the system. Conversely, a lower REC value indicates a sparser or more random structure of the time series.
- Determinism (DET %)—This parameter is used to determine the deterministic structures in the time series. It measures the percentage of points that form diagonal lines of length L, where L is the minimum length of diagonals that are considered deterministic. A larger DET value indicates a greater number of deterministic structures or periodic events in the time series, which can be interpreted as a greater degree of predictability or regularity of the system. Conversely, lower DET values indicate fewer deterministic segments and more randomness in the time series.
- Laminarity (LAM %)—This parameter indicates the percentage of points that form horizontal lines of length w, which is the minimum length of laminated segments that are considered significant. A larger value of LAM indicates a larger number of laminated segments in the time series, which can be interpreted as a greater degree of the duration of a given regime or state of the system and reflects the periodic or steady state in the time series. Conversely, a lower LAM value indicates fewer laminated structures and more randomness in the time series.
- Entropy (ENTR)—This parameter reflects the entropy of the points in the recurrent graph. A higher value of ENTR means greater unpredictability in the time series and greater complexity of the system. In the opposite situation, a lower value of ENTR may indicate greater order or predictability in the time series.

**Phase space reconstruction method**

The phase space reconstruction method is an important tool in the field of non-linear dynamics that can be used to analyze and visualize the dynamics of the studied system based on time series [41]. The main idea of the method is to build a three-dimensional space, the axes of which are related to various parameters and variables to describe the dynamics of the system. This allows the structure and behavior of the system to be visualized and explored in this spatial context. Factors that play an important role in phase space reconstruction are the embedded dimension (d) and the time delay (τ). The false nearest neighbors (FNN) method can be used to determine the embedded dimensionality (d) of data [42,43]. Embedded dimension represents the minimum number of parameters that are needed to describe the internal structure of the data. The first minimum of the average mutual information function (AMI) is a commonly used method for determining the optimal time lag (τ) when constructing time series.

## 3. Results

The study presented in the paper was performed on the records from patients with syncope and arrhythmia, as well as on the records from healthy volunteers. The research was conducted on previously anonymized records. The studies were conducted in the morning, and the patients and healthy volunteers were asked not to drink coffee, not to smoke, not to use alcohol for at least 8 h before conducting the study, and not to consume food in the last 2 h before the examination. The studied individuals did not declare concomitant chronic diseases, were not overweight, were not active athletes, and did not take antidepressants in the days before the study.

The arrhythmia patient cohort was composed of individuals with a diagnosis of extrasystolic arrhythmia. In the Holter records of the examined patients, registered ventricular and supraventricular extrasystoles were found. The basic rhythm of the heart is a sinus rhythm with a minimum heart rate of 60 beats per minute, a maximum of 125 beats per minute, an average for the active period of the day of 85 beats per minute, and for the inactive period 65 beats per minute (these are the average values for the studied records).

The syncope patient cohort was composed of individuals with a diagnosis of vasovagal syncope. According to the information provided by the patients, the cardiac event occurred as a result of emotional stress (in most cases, strong anxiety) accompanied by a prolonged standing position (in the absence of rest). The patients report experiencing dizziness, blurred vision, pallor, sweating, and nausea in the moments before syncope.

Table 1 shows the mean age and gender of the subjects, as well as the calculated standard deviation. There were 44 healthy volunteers (Group 1: 21 men and 23 women; aged 31 to 52 years). In the analyzed total of 48 records of patients diagnosed with arrhythmia (Group 2), 22 were men and 26 were women (aged from 24 to 55 years). Moreover, 42 records with a diagnosis of syncope (Group 3) and a declared age of 26 to 58 years (20 men and 22 women) were analyzed. No significant differences were found between the studied groups of individuals according to these characteristics.

To validate the detection of the QRS complexes of the signals registered with an ECG sensor in the created device, a parallel recording of the ECG was created using an ECG Holter Contec TLC 9803 device (used for long-term monitoring of patients). The examination of the coincidence of the cardio intervals extracted from the two types of studied signals was conducted by determining the relative error and the Mean Squared Error (MSE) by the following formula:

(3) $$MSE=1/N\sqrt{\sum_{i=1}^{N}{\left(x_{i}-y_{i}\right)}^{2}},$$

where:

$x_{i}$—QRS or mean RR intervals (Holter);
$y_{i}$—RR intervals (ECG sensor);
*N*—number of intervals.

The conducted parallel studies on the accuracy of determining the interval time series are summarized in tabular form. Table 2 shows the results for healthy individuals, Table 3 for patients with arrhythmia, and Table 4 for patients with syncope. The values are expressed as mean ± standard deviation (sd).

The following parameters were studied:

- Number of QRS complexes (ECG, Holter record);
- The average value of RR intervals [ms];
- The relative errors ECG/Holter;
- The MSE ECG/Holter.

The conducted studies show that the relative error between the determined QRS complexes (respective RR intervals) with a factory Holter device, as well as for the determined lengths of RR intervals by both methods, are less than 1.5%, which shows that the proposed device can be used for the registration and processing of ECG signals. The studied MSE also has low values.

**Figure 2:**
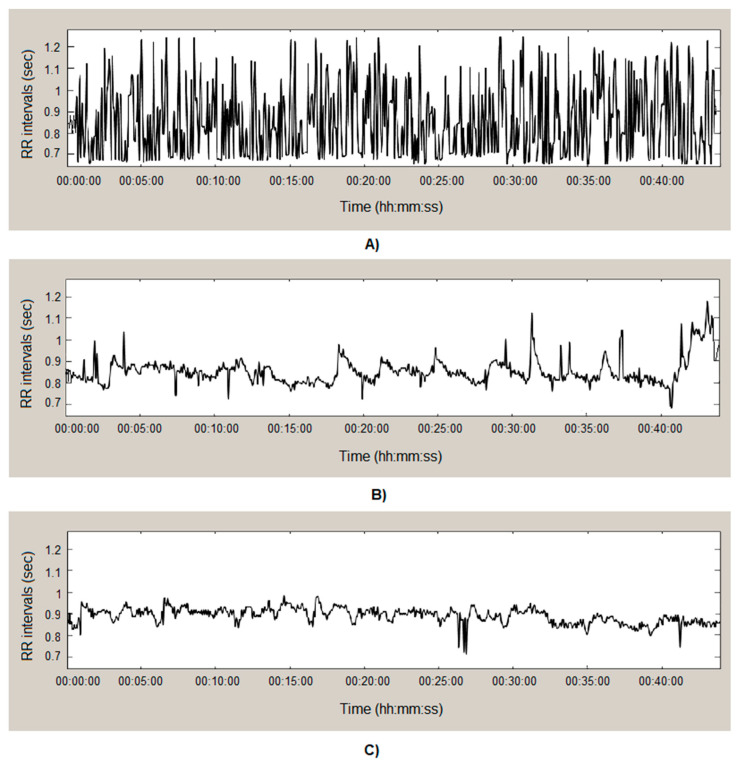
HRV: (**A**) healthy subject; (**B**) patient with arrhythmia; (**C**) patient with syncope.

Figure 2A shows a brief forty-minute section of the studied series in a healthy volunteer. The graph clearly shows the high variability among the consecutive cardiac intervals.

Figure 2B shows a section of an arrhythmia recording. The duration of the interval series varied over a small area, indicating low variability regarding the successive heart intervals.

The recording in Figure 2C is from a recorded cardiac series of a patient with syncope. The variability in the consecutive heart intervals is low, and the adjacent interval values are similar, with few exceptions.

**Figure 3:**
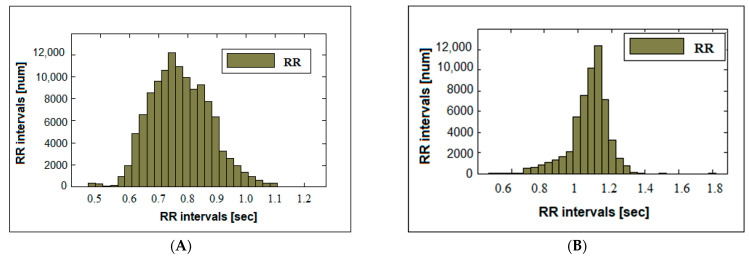
Histograms: (**A**) healthy; (**B**) syncope.

The histogram of the healthy subject (Figure 3A) has the appearance of a normal Gaussian distribution. The distribution of the number of cardiac intervals is central, with the largest percentage of intervals located in a wide range around the central mode of the histogram (with cardiac interval lengths of 0.6 to 1 s). At the same time, there are intervals of different sizes located at both the smaller and larger values of the interval lengths. The histogram of syncope recordings (Figure 3B) has several taller bars and is not symmetrical.

**Figure 4:**
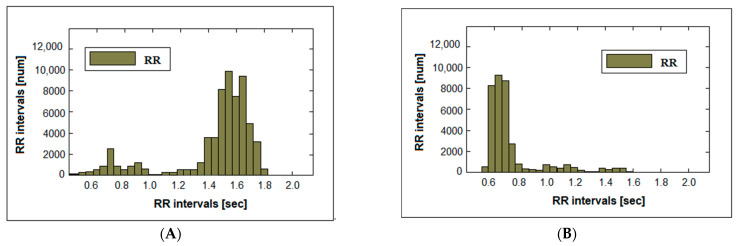
Histograms of patients with arrhythmia: (**A**) sinus bradycardia; (**B**) sinus tachycardia.

The histograms of the arrhythmia recordings are shown in Figure 4. Sinus bradycardia arrhythmia (heart rate > 100 bpm) is shown in Figure 4A; there is a shift in the histogram mode to the right and the presence of a small number of tall bars. Sinus tachycardia-type arrhythmia (heart rate < 60 bpm) is represented in Figure 4B; there is a sharp leftward shift of the histogram mode and a small number (in this case three) of tall bars. The presented histograms show highly nonarrhythmic cardiac activity and concentrations of cardiac burst times in a limited interval.

Table 5 presents the results of the time domain analysis performed on healthy subjects and the two types of records of patients with arrhythmia and syncope. The values are expressed as mean ± standard deviation (sd) or as percentages. A statistical analysis was conducted for the significance of the results via a *t*-Test, comparing the values of the parameters from the healthy group with those of the recordings with arrhythmia and syncope.

The SDNN and SDANN parameters for the arrhythmia and syncope groups were lower compared to the healthy group. The RMSSD parameter was significantly lower in the patients (6.09 ms in arrhythmia and 5.21 ms in syncope) compared to the healthy control group (24.02 ms). The RMSSD provides a very good assessment of the vagus tone, which is also confirmed by other authors’ studies [20,21]. Similar results were observed for the SDNN Index and HRVTi. pNN50 was higher in the patients (49.32 ms arrhythmia and 48.88 ms syncope) compared to the healthy control group (38.08 ms).

The ANOVA method was additionally applied to evaluate the differences between the three groups in the time domain. The comparisons conducted show statistically significant differences between the three studied groups of records (*p*-value < 0.0001) for almost all the studied parameters in the time domain, and, for pNN50, the obtained *p*-value is 0.001 (<0.05, which is the accepted significance level).

Table 6 presents the investigated frequency analysis parameters. A statistical analysis was conducted for the significance of the results via a *t*-test, comparing the values of the parameters from the healthy group with those of the recordings with arrhythmia and syncope.

The ANOVA method was applied to evaluate the differences between the three groups in the frequency domain. The comparisons conducted show statistically significant differences between the three investigated recording groups (*p*-value < 0.0001 for almost all the investigated parameters; 0.007 for LF Power nu and 0.005 for HF Power nu).

The SDNN and SDANN parameters for the arrhythmia and syncope groups were lower compared to the healthy group. The RMSSD parameter was significantly lower in the patients (6.09 ms in arrhythmia and 5.21 ms in syncope) compared to the healthy control group (24.02 ms). The RMSSD provides a very good assessment of the vagus tone, which is also confirmed by other authors’ studies [20,21]. Similar results were observed for the SDNN Index and HRVTi. pNN50 was higher in the patients (49.32 ms arrhythmia and 48.88 ms syncope) compared to the healthy control group (38.08 ms).

Regarding the implementation of a spectral method, in this study, a Welch periodogram was applied using a windowed Hamming function, with a 50% overlap of adjacent windows. The data for the RR intervals were interpolated with a cubic spline basis, and then a 4 Hz sampling rate was applied.

The presented graphical PSDs of the patients and healthy subjects show the spectral distribution of the signal in the three frequency ranges (VLF, LF, and HF) and are plotted in different colors for greater clarity.

**Figure 5:**
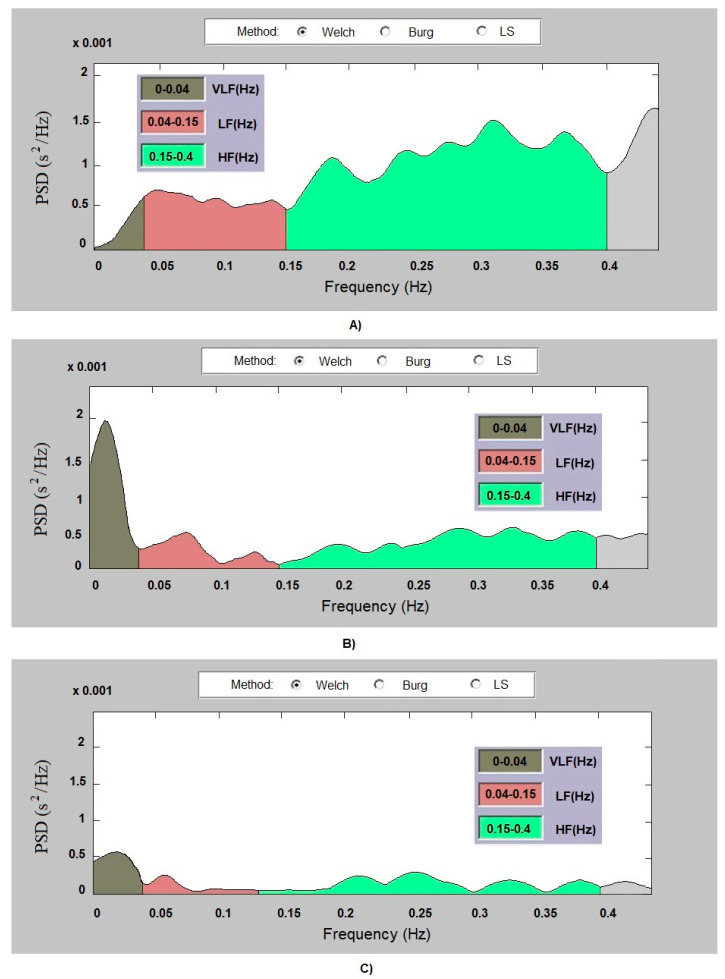
PSD: (**A**) healthy subject; (**B**) patient with arrhythmia; (**C**) patient with syncope.

Figure 5A shows the PSD of a healthy individual obtained by the Welch periodogram method. The PSD values are high in the three ranges tested. The graph shows normal heart rate variability.

Figure 5B shows the PSD of a patient with arrhythmia. The spectrum of the signal is high in the MLF range, while a low spectral density of the signal is observed in the low-frequency and high-frequency ranges. The signal spectrum regarding syncope (Figure 5C) is very low in all the ranges.

The presented graphic results for PSD show that there are differences between the graphic images of the signal power in the individual spectral components in the healthy subjects and subjects suffering from arrhythmia or experiencing syncope. This shows that PSD plots are a good graphical tool for visualizing the life forces of an organism.

**Figure 6:**
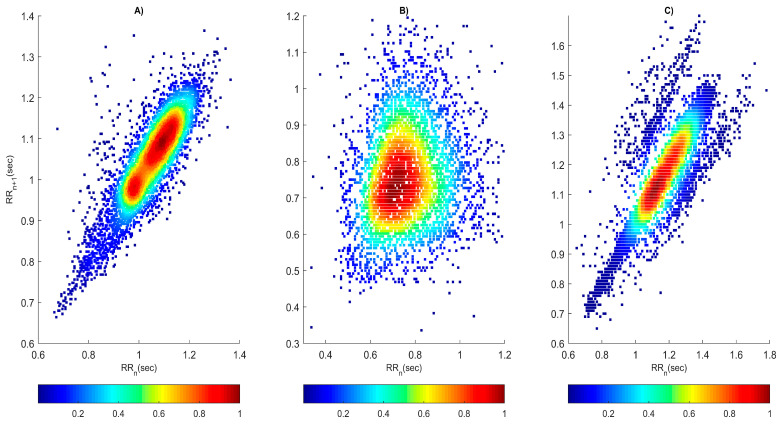
Poincaré plot: (**A**) healthy subject; (**B**) patient with arrhythmia; (**C**) patient with syncope.

The visual analysis of the RR interval series of healthy and diseased individuals, performed using a Poincaré plot, involves the determination of the following three characteristics:

- The shape and distribution of the points in the graph: In healthy individuals, a uniform distribution of the points around the line of identity (the x = y line) is usually observed, which reflects a relatively stable cardiac rhythmicity. In the case of individuals with arrhythmia and syncope, the variations in the points are greater, which may be an indicator of irregularities in the heart rhythm or various problems in the activity of the cardiovascular system. The shapes that the points form in the three types of RR interval series shown in Figure 6 are different. The graph in the healthy individual (Figure 6A) has the shape of a comet, which has a pointed lower part, while, in the patient with arrhythmia, the shape has the appearance of a fan (Figure 6B), and, in the patient with syncope, it has a complex shape consisting of three segments (Figure 6C).
- Asymmetry of points in the plot: From the Poincaré plot, asymmetries in the distribution of points can be observed, which can be an indicator of irregularities in the heart rhythm. There is no asymmetry of points in the observed graphs.
- Scattering of the points on the graph: The scattering of the points can provide information about the degree of heart rate variability. From the graphs shown in Figure 6, the scattering of the points is the smallest in the healthy subject; therefore, the HRV is the largest in him.

The quantitative analysis of the method is determined by the values of the following three parameters: SD1, SD2, and SD1/SD2, which are shown in Table 7. The values of SD1 and SD2 are lower in the patients with arrhythmia and syncope compared to the healthy subjects. Lower values of the SD1 parameter are associated with increased sympathetic tone and less HRV. Higher SD2 values are associated with increased parasympathetic activity and higher HRV. The SD1/SD2 ratio provides information about the balance between sympathetic and parasympathetic activity.

The ANOVA method was applied to evaluate the differences between the three groups using non-linear methods of analysis. The comparisons show statistically significant differences between the three studied groups (*p*-value < 0.0001) for the parameters SD1, SD2, DET, REC, and ENTR. The studied SD1/SD2 ratio did not show statistically significant results.

**Figure 7:**
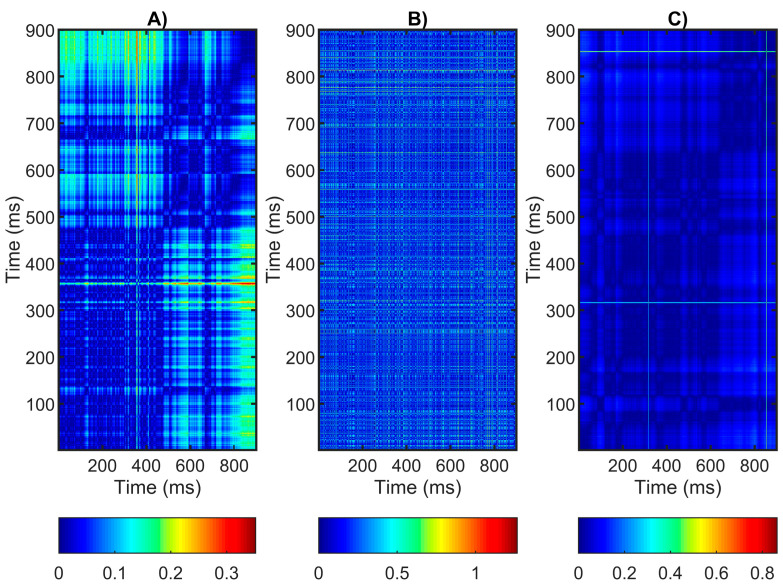
Recurrence plot: (**A**) healthy subject; (**B**) patient with arrhythmia; (**C**) patient with syncope.

The visual analysis of the RR interval series of healthy and diseased individuals by applying a recurrence plot can show the differences in the dynamics between heartbeats and HRV, as well as identify possible abnormalities or disorders in the cardiovascular system. The graphs shown in Figure 7 lack clearly expressed diagonal lines. The graph shown in Figure 7B, corresponding to a patient with arrhythmia, lacks such lines. The absence of diagonal lines in the graph may be due to the following factors:

- The time series does not exhibit obvious periodicity but has chaotic or random behavior where there are no clear repeatable patterns or structures.
- The dominance of the sympathetic or parasympathetic part of the autonomic nervous system on cardiac function.
- The variation in time between the cardiac cycles is less, as is HRV, and this may be reflected in fewer repeating patterns or cycles in the graph.

The horizontal and vertical lines in the graph shown in Figure 7C corresponding to the patient with syncope are less clear, which may be due to the reduced HRV being reflected in fewer periodic or cyclic segments in the graph.

The quantitative analysis of the recurrence plot also provides important information regarding analyzing the dynamics of cardiac activity and in distinguishing between different states of health and disease in individuals. Table 7 shows the results associated with this type of analysis. In the RR intervals of the healthy individuals and sick patients, the values of the REC and DET parameters can have different interpretations and provide information about the characteristics of cardiac activity and the state of the cardiovascular system:

- In the healthy individuals, the REC and DET values are higher than in the patients with arrhythmia and syncope, which is usually associated with stable and regulated cardiac cycles.
- The lower values of REC and DET in the patients with arrhythmia and syncope are associated with cardiac disorders that lead to unstable and unpredictable cardiac cycles that form fewer recurrent structures.

The ENTR parameter of the recurrence plot is a measure of the complexity or unpredictability of the RR interval time series that is embedded in the recurrence structures. A larger value of the ENTR parameter indicates greater unpredictability and greater complexity in the time series, while a smaller value of ENTR indicates less complexity and predictability. In healthy individuals, a higher ENTR value may be associated with greater variation and dynamism in the time series of RR intervals. This reflects the normal physiological fluctuations and reactions of heart activity to various external and internal influences. In sick subjects with arrhythmia and syncope, the ENTR value may be associated with less variation and less dynamism in the time series of RR intervals. This may result from disturbances in cardiac function that lead to more static or maladaptive cardiac activity. A lower ENTR value can be an indicator of the impaired regulation of heart activity and potential problems with the cardiovascular system.

**Figure 8:**
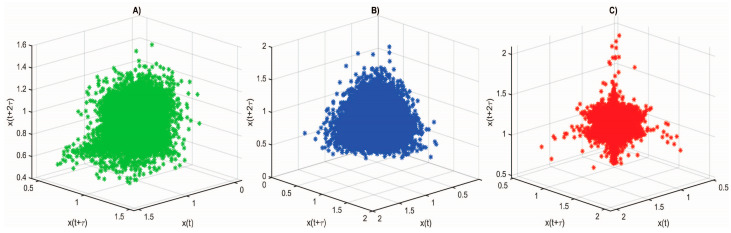
3D phase space attractor for RR intervals: (**A**) healthy subject; (**B**) patient with arrhythmia; (**C**) patient with syncope.

The 3D phase portrait of the attractor of RR signals is a visual representation of the dynamics of cardiac activity in a three-dimensional phase space. It represents the points in the three-dimensional space, each point being defined by the values of three parameters that are selected from the time series of the RR intervals. This attractor is used to analyze the dynamic characteristics of cardiac activity and to distinguish healthy individuals from diseased patients. Figure 8 shows the 3D phase-space attractors for the RR intervals of a healthy individual and of patients with arrhythmia and syncope, which are visually different. In assessing the 3D phase portrait of the attractor of RR signals in diseased and healthy individuals, the following attractor characteristics can be investigated:

- Attractor shape and structure: Healthy and diseased hearts can have different phase portrait shapes and structures. For example, healthy hearts tend to have more regular and organized attractors, while the attractors of patients with heart disease such as arrhythmia and syncope can be more chaotic and irregular.
- Point distribution: The analysis of the point distribution in the phase portrait can provide information on the characteristics of cardiac dynamics. For example, the density and evenness of the distribution may be different between healthy individuals and diseased patients.

**Figure 9:**
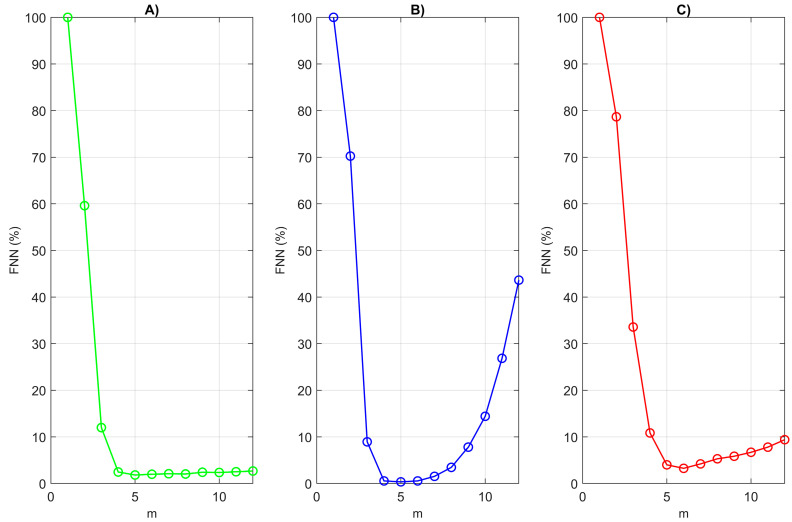
Embedding dimension (m) determined by the FNN method for RR intervals: (**A**) healthy subject; (**B**) patient with arrhythmia; (**C**) patient with syncope.

An important element in the study of RR interval series is the reconstruction of the attractor. The evaluation of the 3D phase portrait of the attractor of the RR signals in diseased and healthy subjects can provide information on the differences in the cardiac activity dynamics between the two types of subjects. The FNN relationship is a statistical method for estimating the dimensionality of the phase attractor that can be applied to the study of non-linear dynamical systems. Figure 9 illustrates the results of the estimation of the dimension of the attractor reconstruction for the RR intervals for healthy individuals and for patients with arrhythmia and syncope, showing the dependence regarding the number of false nearest neighbors (%) on the embedded dimension (m). It can be seen from the figure that the number of false nearest neighbors decreases sharply at the reconstruction size m = 4 in a healthy individual, in a patient with arrhythmia, and in a patient with syncope m = 5. An increase in the number of false nearest neighbors was observed in the patients with arrhythmia and syncope at m > 4.

**Figure 10:**
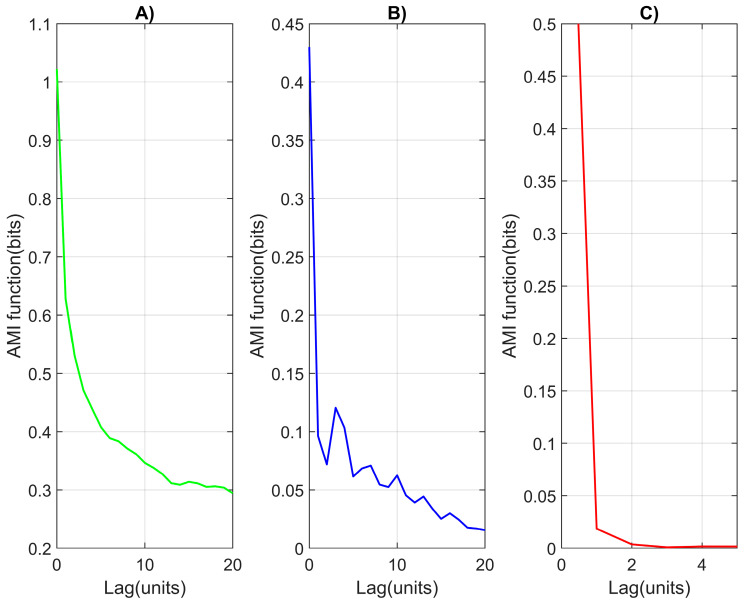
Relationship between time lag and the AMI function for RR intervals: (**A**) healthy subject; (**B**) patient with arrhythmia; (**C**) patient with syncope.

Figure 10 shows the determination of the optimal delay value of the RR intervals by using the first minimum of the AMI function of the three studied subjects. The first minimum of the AMI function is fifteen for a healthy subject, three for an arrhythmia patient, and two for a syncope patient.

## 4. Discussion

The presented research demonstrates how the mathematical methods for HRV analysis can be correctly performed thanks to the successful registration method and the effectively conducted preprocessing of the signals in the created mobile device. Essential to conducting accurate HRV analysis is thorough preprocessing, including appropriate methods to remove artifacts and other signal disturbances.

The parameters in the time domain provide a very good estimate of the cardiac activity in the denoised signals. If, despite the noise reduction procedures, the signals have high noise levels, then the geometric indices HRVTi and TINN provide a good estimate even in the presence of noise.

The frequency parameters provide an opportunity to obtain the correct idea of the action of the cardiovascular system using a quantitative evaluation of the mechanism of the autonomous control of the nervous system. The development of HRV methods shows the presence of a stable relationship between the signal power in the high frequency range and vagal tone. Combining the evaluation in the frequency domain with similar parameters in the time domain (for example, the RMSSD and pNN50 parameters correspond to the HF spectrum in the time domain) enables a more precise interpretation of the obtained results.

When evaluating HRV parameters, it is good to take into account the age range of the particular subject as there are differences in heart rate between average individuals and athletes, for example, as well as between children, middle-aged people, and the elderly.

The visual and quantitative evaluation of the RR interval series obtained by the non-linear methods, such as the Poincaré plot and recurrence plot, can provide valuable information about the dynamics and characteristics of the cardiac activity of the studied subjects, which can be useful in the diagnosis or monitoring of patients with various cardiovascular diseases and functional conditions. The Poincaré plot is a method for visualizing the correlation between successive intervals in the RR time series and can be used to assess the heart rate variability and cyclicity. The shape and distribution of the points in a Poincaré plot can be used to assess the degree of regularity and stability of cardiac activity. Our experimental results show that, in healthy people, the graph has a comet shape, while, in arrhythmia, it looks like a fan, and, in syncope, it has a complex shape consisting of three segments. Similar results have been reported in other publications [22,26]. A recurrence plot is a graphical method of visualizing the recurring structures in the time series of RR intervals and can be used to analyze the stability and regularity of the heart rhythm. Different characteristics of the recurrence plot, such as the length and number of diagonal lines, can be analyzed to assess the dynamics of cardiac activity. Our graphical results show that, in healthy subjects, the graph consists of fewer squares, indicating higher HRV, and, in sick subjects (arrhythmia and syncope), the graphs have more squares, indicating signal periodicity and lower HRV. Similar results are shown in another publication [30]. Combining the recurrence plot and the Poincaré plot can complement their mutual understanding and provide a more complete picture of an individual’s cardiac function. The interrelationships between the structures observed in the recurrence plot and the correlations detected in the Poincaré plot can be explored, which can lead to a deeper understanding of the dynamics of cardiac activity. The relationship between the recurrence plot and the Poincaré plot regarding the RR intervals on the one hand and HRV on the other hand is indicative of the reality that the graphs constructed with these two methods reflect the structural and dynamic characteristics of the time series that can conduct an assessment regarding HRV and the state of the cardiovascular system. In this way, new knowledge and possibilities for the diagnosis and monitoring of various cardiovascular diseases can be discovered. In addition to the information obtained with these two non-linear visual methods, the evaluation of the 3D phase portrait of the attractor of the RR signals can also provide important information about the dynamics of cardiac activity and the differences between healthy and diseased individuals, which can create a more complete image of the cardiovascular activity. The statistical analysis, in addition to visual and quantitative analyses with the application of the Poincaré plot and recurrence plot, proved that five of the six parameters studied have statistical significance and can be used to distinguish healthy individuals from patients with arrhythmia and syncope.

The comprehensive analysis of the HRV parameters presented enables the interpretation of the results and discussion of their applicability, utility, and limitations.

The completed research has certain limitations related to the number of records examined. The work in this direction will continue in terms of registering and examining new records.

The authors will direct their future research toward differentiating different types of arrhythmias and different types of syncope. For this purpose, the long-term observation of a larger number of patients diagnosed with these diseases is necessary.

## 5. Conclusions

HRV shows disturbances regarding the autonomic nervous system activity in patients with arrhythmia and syncope. A decrease in the HRV parameters was observed most often. The creation and use of mobile, light, and convenient devices for recording ECG and PPG signals enable the monitoring, analysis, and assessment of the HRV in real-life conditions of individuals, which will contribute to preserving and improving the health status of individuals, fast and adequate medical intervention (urgent care for life-threatening conditions), effective recovery, and ensuring high quality of life.

## Figures and Tables

**Table 1:** Participants’ demographic characteristics.

Parameter	Group 1 *N* = 44	Group 2 *N* = 48	Group 3 *N* = 42	*p*-Value	
				Gr1/Gr2	Gr1/Gr3
Gender, Men [%]	47.73	48.83	47.62	NS ^1^	NS ^1^
Age ± sd	45.82 ± 20.14	47.98 ± 17.22	48.33 ± 18.39	NS ^1^	NS ^1^

^1^ Not significant.

**Table 2:** Table of defined parameters for the two signals for healthy individuals.

Parameters	ECG *N* = 44 [Mean ± sd]	Holter N=48 [Mean ± sd]	Relative Error [%] ECG/Holter	MSE ECG/Holter
Number of QRS complexes per 2 h	8593.54 ± 322.12	8639.09 ± 465.28	0.49%	0.0115
Mean RR intervals [ms]	839.88 ± 134.52	834.54 ± 126.65	0.47%	0.012

**Table 3:** Table of defined parameters for the two types of signals for patients with arrhythmia.

Parameters	ECG *N* = 48 [Mean ± sd]	Holter N=48 [Mean ± sd]	Relative Error [%] ECG/Holter	MSE ECG/Holter
Number of QRS complexes/pulse waves per 1 h	9634.58 ± 528.04	9707.18 ± 532.63	0.75%	0.019
Mean RR intervals [ms]	748.29 ± 125.57	742.06 ± 137.61	0.81%	0.0185

**Table 4:** Table of defined parameters for the two types of signals for patients with syncope.

Parameters	ECG *N* = 42 [Mean ± sd]	Holter N=48 [Mean ± sd]	Relative Error [%] ECG/Holter	MSE ECG/Holter
Number of QRS complexes/pulse waves per 1 h	10,112.58 ± 481.36	10,236.37 ± 443.92	1.21%	0.035
Mean RR intervals [ms]	712.33 ± 114.61	703.52 ± 123.82	1.28%	0.041

**Table 5:** Time domain parameters.

Parameters	Group 1 *N* = 44 [Mean ± sd]	Group 2 *N* = 48 [Mean ± sd]	Group 3 *N* = 42 [Mean ± sd]	*p*-Value (*t*-Test)		*p*-Value (ANOVA)
				Gr1/Gr2	Gr1/Gr3	Gr1/Gr2/Gr3
MeanRR (ms)	892.41 ± 117.12	745.62 ± 131.23	654.14 ± 136.09	<0.0001	<0.0001	<0.0001
SDNN (ms)	151.08 ± 43.72	94.81 ± 63.08	82.01 ± 92.36	<0.001	<0.001	<0.001
SDANN (ms)	134.35 ± 82.39	82.09 ± 51.06	74.11 ± 13.62	<0.001	<0.001	<0.001
RMSSD (ms)	24.02 ± 11.23	6.09 ± 5.86	5.21 ± 3.24	<0.0001	<0.001	<0.001
pNN50	32.14 ± 18.22	44.27 ± 23.67	49.54 ± 36.83	<0.001	<0.005	<0.05
SDNN Index (ms)	81.41 ± 42.05	49.32 ± 36.04	48.88 ± 34.19	<0.005	<0.005	<0.005
HRVTi	34.08 ± 22.33	21.34 ± 20.53	14.42 ± 12.76	<0.01	<0.0001	<0.001
TINN [ms]	522.35 ± 318.01	455.61 ± 203.88	309.19 ± 22.94	NS ^1^	<0.001	NS ^1^

^1^ NS—not significant.

**Table 6:** Frequency analysis parameters.

Parameters	Group 1 *n* = 44 [Mean ± sd]	Group 2 *n* = 48 [Mean ± sd]	Group 3 *n* = 42 [Mean ± sd]	*p*-Value (*t*-Test)		*p*-Value (ANOVA)
				Gr1/Gr2	Gr1/Gr3	Gr1/Gr2/Gr3
VLF Power [ms^2^]	3431.38 ± 842.86	8327.35 ± 948.72	2641.06 ± 242.02	<0.0001	<0.0001	<0.0001
LF Power [ms^2^]	1432.44 ± 498.03	798.36 ± 141.52	461.95 ± 112.62	<0.0001	<0.0001	<0.0001
HF Power [ms^2^]	849.12 ± 278.02	581.18 ± 188.72	442.76 ± 268.35	<0.0001	<0.0001	<0.0001
LF Power nu	0.63 ± 0.11	0.58 ± 0.12	0.51 ± 0.19	<0.05	<0.001	0.007
HF Power nu	0.37 ± 0.09	0.42 ± 0.12	0.49 ± 0.22	<0.05	<0.05	0.005
LF/HF	1.69 ± 0.43	1.37 ± 0.46	1.04 ± 0.61	<0.001	<0.0001	<0.001

**Table 7:** Quantitative analysis, by Poincaré plot and recurrence plot, of three types of RR interval series.

Parameter	Group 1 *n* = 44 [Mean ± sd]	Group 2 *n* = 48 [Mean ± sd]	Group 3 *n* = 42 [Mean ± sd]	*p*-Value (*t*-Test)		*p*-Value (ANOVA)
				Gr1/Gr2	Gr1/Gr3	Gr1/Gr2/Gr3
Poincaré plot						
SD1 [ms]	66.12 ± 9.12	45.51 ± 10.22	41.32 ± 11.67	0.0001	0.0001	0.0001
SD2 [ms]	232.34 ± 29.23	148.12 ± 25.36	140.44 ± 21.77	0.0001	0.0001	0.0001
SD1/SD2 [-]	0.31 ± 0.09	0.29 ± 0.15	0.27 ± 0.21	0.4452	0.6012	0.67
Recurrence plot						
DET [%]	90.8 ± 5.14	97.08 ± 6.13	99.34 ± 11.41	0.0001	0.0001	0.0001
REC [%]	36.43 ± 1.23	44.41 ± 1.96	47.24 ± 2.11	0.0001	0.0001	0.0001
ENTR [-]	4.13 ± 0.18	3.58 ± 0.41	3.21 ± 0.31	0.0001	0.0001	0.0001

## Data Availability

The cardio data we processed for the research purposes of this paper were obtained from the Medical University of Varna, Bulgaria (available at http://hrvdata.vtlab.eu/, (accessed on 12 March 2024)).

## References

[1] Xie Y., Xu E., Bowe B., Al-Aly Z. (2022). Long-term cardiovascular outcomes of COVID-19. Nat. Med..

[2] Vosko I., Zirlik A., Bugger H. (2023). Impact of COVID-19 on Cardiovascular Disease. Viruses.

[3] Acharya U.R., Ghista D., Yi Z., Min L., Ng E., Sree S., Faust O., Weidong L., Alvin A. Integrated index for cardiac arrhythmias diagnosis using entropies as features of heart rate variability signal. Proceedings of the 2011 1st Middle East Conference on Biomedical Engineering.

[4] Jose S.K., Wayanad, Shambharkar C.M., Chunkath J. Cardiac arrhythmia detection using ballistocardiogram signal. Proceedings of the 2015 IEEE International Conference on Signal Processing, Informatics, Communication and Energy Systems (SPICES).

[5] Reed M.J., Robertson C.E., Addison P.S. (2005). Heart rate variability measurements and the prediction of ventricular arrhythmias. QJM Int. J. Med..

[6] Inan O.T., Giovangrandi L., Kovacs G.T.A. (2006). Robust neural-network based classification of premature ventricular contractions using wavelet transform and timing interval features. IEEE Trans. Biomed. Eng..

[7] Ivanova V., Boneva A., Ivanov S., Doshev Y. (2023). An ECG Monitoring Device for a Modular Instrument to Surgical Robots. Proceedings of the XXXII International Scientific and Technical Conference Automation of Discrete Production Engineering—ADP 2023.

[8] Malik M. (1996). Task Force of the European Society of Cardiology and the North American Society of Pacing and Electrophysiology, Heart rate variability—Standards of measurement, physiological interpretation, and clinical use. Circulation.

[9] Nayak S.K., Pradhan B., Mohanty B., Sivaraman J., Ray S.S., Wawrzyniak J., Jarzębski M., Pal K. (2023). A Review of Methods and Applications for a Heart Rate Variability Analysis. Algorithms.

[10] Kim H.G., Cheon E.J., Bai D.S., Lee Y.H., Koo B.H. (2018). Stress and heart rate variability: A meta-analysis and review of the literature. Psychiatry Investig..

[11] Muhajir D., Mahananto F., Sani N.A. (2022). Stress level measurements using heart rate variability analysis on android based application. Sixth Information Systems International Conference (ISICO 2021). Procedia Comput. Sci..

[12] Scherer M., Martinek J., Mayr W. (2019). HRV (Heart Rate Variability) as a non-invasive measurement method for performance diagnostics and training control. Curr. Dir. Biomed. Eng..

[13] Mosley E., Laborde S. A scoping review of heart rate variability in sport and exercise psychology. Int. Rev. Sport Exerc. Psychol..

[14] Burlacu A., Brinza C., Brezulianu A., Covic A. (2021). Accurate and early detection of sleepiness, fatigue and stress levels in drivers through Heart Rate Variability parameters: A systematic review. Rev. Cardiovasc. Med..

[15] Ishaque S., Khan N., Krishnan S. (2021). Trends in heart-rate variability signal analysis. Front. Digit. Health.

[16] (2021). *COVID-19 and the Heart: What Have We Learned?*; Harvard Health Publishing: Harvard Medical School, Cambridge, MA, USA. https://www.health.harvard.edu/blog/covid-19-and-the-heart-what-have-we-learned-2021010621603.

[17] Suh H.W., Kwon C.Y., Lee B. (2023). Long-Term Impact of COVID-19 on Heart Rate Variability: A Systematic Review of Observational Studies. Healthcare.

[18] Akar S.A., Kara S., Latifoglu F., Biggic V. (2013). Spectral analysis of photoplethysmographic signals: The importance of preprocessing. Biomed. Signal Process. Control..

[19] Paniccia M., Paniccia D., Thomas S., Taha T., Reed N. (2017). Clinical and non-clinical depression and anxiety in young people: A scoping review on heart rate variability. Auton Neurosci..

[20] Pham T., Lau Z.J., Chen S.A., Makowski D. (2021). Heart Rate Variability in Psychology: A Review of HRV Indices and an Analysis Tutorial. Sensors.

[21] Laborde S., Mosley E., Thayer J.F. (2017). Heart rate variability and cardiac vagal tone in psychophysiological research–recommendations for experiment planning, data analysis, and data reporting. Front. Psychol..

[22] Ernst G. (2014). Heart Rate Variability.

[23] Gomes R.L., Vanderlei L., Garner D.M., Santana M.D., Abreu L.C., Valenti V.E. (2018). Poincaré plot analysis of ultra-short-term heart rate variability during recovery from exercise in physically active men. J. Sports Med. Phys. Fit..

[24] Fishman M., Jacono F.J., Park S., Jamasebi R., Thungtong A., Loparo K.A., Dick T.E. (2012). A method for analyzing temporal patterns of variability of a time series from Poincare plots. J. Appl. Physiol..

[25] Kamen P., Krum H., Maxwell A. (1996). Poincaré plot of heart rate variability allows quantitative display of parasympathetic nervous activity in humans. Clin. Sci..

[26] Khandoker A.H., Karmakar C., Brennan M., Palaniswami M., Voss A. (2013). Poincaré Plot Methods for Heart Rate Variability Analysis.

[27] Hoffmann B., Flatt A.A., Silva L.E.V., Młyńczak M., Baranowski R., Dziedzic E., Werner B., Gąsior J.S. (2020). A Pilot Study of the Reliability and Agreement of Heart Rate, Respiratory Rate and Short-Term Heart Rate Variability in Elite Modern Pentathlon Athletes. Diagnostics.

[28] Urzeală C., Bota A., Serbanoiu S., Mezei M., Dutheil F., Courteix D. (2020). 2020. Heart Rate Variability as a Possible Predictor of Sport Performance in Junior Rhythmic Gymnastics. Isokinet. Exerc. Sci..

[29] Blásquez J.C.C., Font G.R., Ortís L.C. (2009). 2009. Heart-rate variability and precompetitive anxiety in swimmers. Psicothema.

[30] Acharya U.R., Suri J.S., Spaan J.A.E., Krishnan S.M. (2007). Advances in Cardiac Signal Processing.

[31] Lebamovski P.D. (2022). Impact of Stress on Heart Rate Variability. Proc. CBU Med. Pharm..

[32] Lebamovski P.D. (2023). The Influence of Virtual Reality on the Autonomic Nervous System.

[33] Lebamovski P.D. (2022). Analysis of Methods and Approaches for Evaluation of Heart Rate Variability.

[34] Ivan C., Arva M.C. (2022). Nonlinear Time Series Analysis in Unstable Periodic Orbits Identification-Control Methods of Nonlinear Systems. Electronics.

[35] Mathunjwa B.M., Lin Y.-T., Lin C.-H., Abbod M.F., Sadrawi M., Shieh J.-S. (2022). ECG Recurrence Plot-Based Arrhythmia Classification Using Two-Dimensional Deep Residual CNN Features. Sensors.

[36] Zhou C., Zhang W. (2015). Recurrence Plot Based Damage Detection Method by Integrating Control Chart. Entropy.

[37] Petrauskiene V., Pal M., Cao M., Wang J., Ragulskis M. (2022). Color Recurrence Plots for Bearing Fault Diagnosis. Sensors.

[38] Hanáková L., Průcha J., Socha V., Štengl M., Van den Bergh S. (2020). Effect of High-Induction Magnetic Stimulation on Complex Heart Rate Variability of Sus Scrofa Domesticus under General Anesthesia. Appl. Sci..

[39] Zimatore G., Gallotta M.C., Campanella M., Skarzynski P.H., Maulucci G., Serantoni C., De Spirito M., Curzi D., Guidetti L., Baldari C. (2022). Detecting Metabolic Thresholds from Nonlinear Analysis of Heart Rate Time Series: A Review. Int. J. Environ. Res. Public Health.

[40] Calderón-Juárez M., González-Gómez G.H., Echeverría J.C., Pérez-Grovas H., Lerma C. (2020). Association between Mean Heart Rate and Recurrence Quantification Analysis of Heart Rate Variability in End-Stage Renal Disease. Entropy.

[41] Błażkiewicz M. (2022). Evaluation of Geometric Attractor Structure and Recurrence Analysis in Professional Dancers. Entropy.

[42] Nayak S.K., Bit A., Dey A., Mohapatra B., Pal K. (2018). A Review on the Nonlinear Dynamical System Analysis of Electrocardiogram Signal. J. Healthc. Eng..

[43] Krakovská A., Mezeiová K., Budáčová H. (2015). Use of false nearest neighbors for selecting variables and embedding parameters for state space reconstruction. J. Complex Syst..

